# Dexmedetomidine versus propofol for sedation in patients undergoing vitreoretinal surgery under sub-Tenon’s anesthesia

**DOI:** 10.4103/1658-354X.76506

**Published:** 2011

**Authors:** Ashraf Ghali, Abdul Kader Mahfouz, Tapio Ihanamäki, Ashraf M. El Btarny

**Affiliations:** *Department of Anaesthesiology, Magrabi Eye & Ear Hospital, Muscat, Sultanate of Oman*; 1 *Department of Anaesthesiology, Al Nahda Hospital, Ministry of Health, Muscat, Sultanate of Oman*; 2 *Department of Ophthalmology, Helsinki University, Central Hospital, Helsinki, Finland and Magrabi Eye & Ear Hospital, Muscat, Sultanate of Oman*; 3 *Department of Ophthalmology, Magrabi Eye & Ear Hospital, Muscat, Sultanate of Oman*

**Keywords:** *Dexmedetomidine*, *propofol*, *sedation*, *vitreoretinal surgery*

## Abstract

**Purpose:**

The purpose of this study was to evaluate the hemodynamic, respiratory effects, the recovery profile, surgeons, and patients satisfaction with dexmedetomidine sedation compared with those of propofol sedation in patients undergoing vitreoretinal surgery under sub-Tenon’s anesthesia.

**Methods:**

Sixty patients were enrolled in this prospective, single-blind, randomized study. The patients were divided into two groups to receive either dexmedetomidine (group D) or propofol (group P). Sedation level was titrated to a Ramsay sedation scale (RSS) of 3. Hemodynamic and respiratory effects, postoperative recovery time, analgesic effects, surgeons and patients satisfaction were assessed.

**Results:**

Both groups provided a similar significant reduction in heart rate and mean arterial pressure compared with baseline values. The respiratory rate values of the dexmedetomidine group were significantly higher than those in the propofol group. The oxygen saturation values of the dexmedetomidine group were significantly higher than those of the propofol group. The expired CO_2_ was similar in both groups. Postoperatively, the time to achieve an Aldrete score of 10 was similar in both groups. Dexmedetomidine patients have significantly lower visual analog scale for pain than propofol patients. The surgeon satisfaction with patients’ sedation was similar for both groups. The patients’ satisfaction was higher in the dexmedetomidine group.

**Conclusion:**

Dexmedetomidine at similar sedation levels with propofol was associated with equivalent hemodynamic effects, maintaining an adequate respiratory function, similar time of discharge from PACU, better analgesic properties, similar surgeon’s satisfaction, and higher patient’s satisfaction. Thus, dexmedetomidine may prove to be a valuable adjuvant for sedation in patients undergoing vitreoretinal surgery under sub-Tenon’s anesthesia.

## INTRODUCTION

For many ophthalmic surgeons, local anesthesia (LA) has become the preferred option over general anesthesia (GA) because of quicker patient rehabilitation and the avoidance of possible complications from general anesthesia.[1] Several methods of LA for vitreoretinal (VR) cases have been described, including retrobulbar, peribulbar, sub-Tenon’s, and even topical anesthesia in some cases.[2] Many drugs have been used for sedation during eye surgery like, propofol, benzodiazepines, and opioids, with a relative risk of oversedation and disorientation, confusion, or increased risk of respiratory depression and oxygen desaturation. All of these untoward effects may hamper patients’ cooperation during surgery, and would make these agents less than ideal for the intraoperative management of sedation.[3] Propofol is widely used for sedation during eye surgery because of its short duration of action, no cumulative effect, unique recovery profile as well as its rapid emergence.[2] In contrast, dexmedetomidine is a highly selective alpha-2-adrenoreceptor agonist with both sedative and analgesic properties and is devoid of respiratory depressant effect.[4] Dexmedetomidine has been studied for sedationand analgesia sparing properties in surgical settings[3] but not in vitreoretinal surgery. Therefore, the purpose of this study was to evaluate the hemodynamic and respiratory effects (as a primary end-point) and the recovery profile and surgeons and patients satisfaction (as a secondary end-point) with dexmedetomidine sedation compared with those of propofol sedation in patients undergoing vitreoretinal surgery under sub-Tenon’s anesthesia.

## METHODS

After obtaining approval from the Institutional Ethics Committee and written informed consent from all patients, 60 adult patients (ASA I-III) of both sexes were scheduled for retinal detachment surgery including pneumatic retinopexy, segmental buckling±gas, and vitrectomy±gas±silicone oil. Vitrectomy for epiretinal membrane or macular hole was included. The patients were enrolled in this prospective, single-blind, randomized study in case that the expected time of surgery to be less than 2 h. Exclusion criteria included age younger than 18 years, the usual contraindications for regional anesthesia such as patients refusing LA, clotting abnormalities, impaired mental status, or allergy to any of the study medications. Also, patients were excluded if they had severe cardiac disease, chronic obstructive lung disease, and a history of sleep apnea. This study was carried out in the Magrabi Eye and Ear Hospital in Oman by the January 2008 to December 2009. Patients were randomly (the block randomization method was used with the block size determined to be six) allocated to one of the two groups to receive either dexmedetomidine (group D, *n*=30) or propofol (group P, *n*=30) for sedation during surgery under sub-Tenon’s anesthesia. The patients were masked to the treatment arms. All measures were assessed by the first author, while the perioperative anesthesia management and drug preparation were performed by the second author. All operations were performed by the same surgeon (fourth author) who was blinded to the technique of sedation.

Patients arrived in the operating room fasted for 8 h and unpremedicated, a peripheral i.v. catheter was inserted and standard monitoring, including noninvasive arterial blood pressure (MAP), electrocardiogram (5 leads), heart rate (HR), and peripheral oxygen saturation (SpaO_2_) were used. A nasal cannula was applied and supplemental oxygen was given throughout the procedure at 2 L/min. Expired CO_2_ (infrared spectroscopy) was sampled from one port of the cannula. Sedation level was assessed every 5 min by using the Ramsay sedation scale (RSS)[5] which was explained to the patients during the preoperative visit. Dexmedetomidine (Precedex, 200 μg per 2 mL; Abbott, USA) was diluted with 0.9% NaCl to a concentration of 4 μg/mL in 50 ml syringe. Group D patients received dexmedetomidine1 μg/kg i.v. over 10 min using an infusion pump (AS50TM, Baxter Health Care Co., Singapore), and followed by a continuous infusion of dexmedetomidine 0.2 to 0.6 μg/kg /h, starting at 0.4 μg/kg/h and titrated every 5 min, in steps of 0.1 μg/kg/h. In Group P, an initial dose of propofol (propofol 1% fresenius, contains: 10 mg/mL propofol) was infused i.v. over 10 min at 0.7 mg/kg, followed by a maintenance infusion of 0.5 to 2 mg/kg/h (the propofol doses are adjusted from our experience in the hospital for sedation during ophthalmic regional anesthesia). To achieve adequate sedation in both groups, infusion doses of test drugs were titrated as required to achieve the target RSS of 3 [Table 1]. During the procedure, if bradypnea (RR<10) or SpaO_2_ was 92% or less, bradycardia (HR<45) and hypotension (MAP<50) were recorded, 4 L/min of supplemental oxygen was administered via a nasal cannula, 0.5 mg atropine was administered, and 0.9% saline was infused, respectively, with reducing rate of infusion of the drug aiming to awake the patient and to resume his normal breath. The infusion pump was stopped at the end of the procedure in both groups.

**Table 1:** Ramsay sedation score

Score	Observation
1	Anxious, agitated or restless
2	Cooperative, oriented and tranquil
3	Responsive to commands
4	Asleep, but with brisk response to light glabellar tap or loud auditory stimulus
5	Asleep, sluggish response to glabellar tap or auditory stimulus
6	Asleep, no response

After completing the loading dose of the study drug, sub-Tenon’s block was performed. After instilling benoxinate hydrochloride (Novesin 0.4%, Novartis, Switzerland) eye drops, a 2 mm radial conjunctival and tenon capsule incision down to bare sclera was done, starting approximately 6 mm from the limbus in the inferotemporal quadrant. Through it, a blunt cannula was placed in the episcleral space and toward the retrobulbar space, where a 5 mL of the local anesthetic solution (0.75% ropivacaine plus hyaluronidase 15 IU/ mL) was injected.[6]

**Figure 1:**
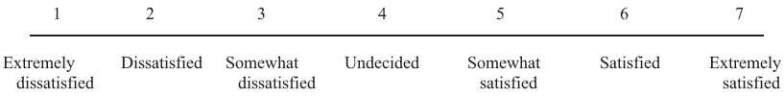
A 7-point Likert-like verbal rating scale for assessment of patients’ satisfaction with intraoperative sedation/analgesia

The following measures were assessed:

1. The time to achieve adequate sedation level was documented (time from start of infusion of the study drug till achieving the target sedation level).
2. Heart rate, mean arterial pressure, respiratory rate (RR), oxygen saturation, and expired CO_2_ were recorded every 5 min throughout the surgery and in the immediate postoperative period (at 15 and 30 min).
3. In the recovery room, Aldrete score[7] was determined every 5 min until discharge. Patients were deemed ready for discharge when they had achieved an Aldrete score of 10.
4. The degree of pain was assessed by using a 10-cm visual analog scale for pain where: 0=no pain and 10=intolerable pain at 1, 2, 3, 4, 5, and 6 hours after the end of surgery.
5. Patients were asked to answer the question ‘How would you rate your experience with the sedation/analgesia you have received during surgery?’ using a 7-point Likert-like verbal rating scale[8] [Figure 1]. This assessment of patients’ satisfaction with sedation and analgesia was performed at 6 h after the end of surgery.
6. The surgeon was asked to rate his satisfaction with patient sedation using the same method and scale at the end of surgery.
7. All adverse events including, but not limited to, bradycardia (HR<45 beats/min), hypotension (MAP<50 mmHg sustained for >10 min), respiratory depression (RR<10 bpm), or oxygen desaturation (SpaO_2_ <92%) were recorded.

### Statistical analysis

The number of patients was determined on the basis of the results of a preliminary investigation during which the sample size was calculated to be30 patients per group based on the reduction in heart rate in both groups during the sedation period as the primary endpoint, a population variance of (2)^2^, a two-sided α of 0.05, and a power of 90%. Sample Size Calculations Program version 2.1.31 (Copyright^©^ 1997 by WD DuPont and WD Plummer) was used. The statistical analysis of our results was conducted using the computer program SPSS version 15.0 for Windows (SPSS Inc., Chicago, IL, USA). Data were expressed as mean±SD. The two way repeated measures ANOVA was used to compare the interval data, and *post hoc* Tukey test was used as the *post hoc* test to determine differences between and within groups. We considered *P*<0.05 to be statistically significant.

## RESULTS

**Figure 2:**
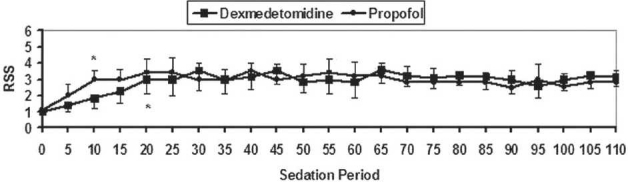
Levels of RSS during the intraoperative period (sedation period). Data are displayed as means±standard deviations. *Statistically significant compared to the baseline value. †Statistically significant compared to group D.

**Figure 3:**
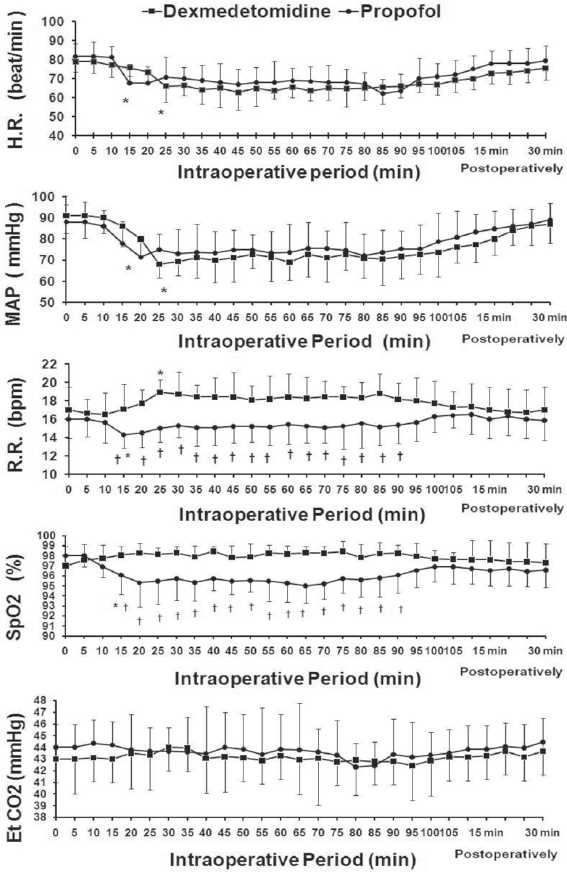
Cardio-respiratory changes during the intraoperative period and the immediate postoperative period. Time (0) is the start of study drug administration. Data are displayed as means±standard deviations. * Statistically significant compared to the baseline value. † Statistically significant compared to group D.

The two groups were comparable with respect to the following variables; age, sex, weight, ASA status, and duration of surgery (*P*>0.05). The time required from the start of the infusion of the study drugs to achieve targeted levels of sedation was significantly longer in the dexmedetomidine group (20.36±4.66 min) than in the propofol group (10.96±3.27 min) (*P*=0.001) [Table 2]. However, there was no significant difference in the RSS levels throughout the sedation period in both groups [Figure 2]. In both groups, there were a similar significant reduction in HR and MAP compared with baseline values (*P*>0.05). Furthermore, the RR values in the dexmedetomidine group were significantly increased (*P*<0.05) compared with baseline values, while there was significant reduction in the RR in the propofol group (*P*<0.05) compared with baseline values. RR values in the dexmedetomidine group were significantly higher than those in the propofol group during the sedation period (*P*<0.05). The SpaO_2_ values in the dexmedetomidine group did not change from baseline, while there was significant reduction in the SpaO_2_ in the propofol group (*P*<0.05) compared with the baseline values. SpaO_2_ values in the dexmedetomidine group were significantly higher than those in the propofol group during the sedation period (*P*<0.05). The expired CO_2_ was similar in both groups (*P*>0.05) [Figure 3]. In the immediate postoperative period, all the cardiorespiratory measures returned back to the normal preoperative values within 15 min. Nevertheless, there were no episodes of bradycardia, hypotension, desaturation, nausea and vomiting, or dry mouth in either group.

**Table 2:** Demographic and selected clinical data of the study groups

	Group D (n=30)	Group P (n=30)	*P* value
Age (yr)	49.16±4.47	47.10±8.02	0.337
Weight (kg)	73.00±4.74	70.36±5.89	0.062
Sex (M/F)	13/17	14/16	0.792
ASA class I/II/III (n)	14/12/ 4	13/11/ 6	0.1
Duration of surgery (min)	102.03±7.62	99 73±6.29	0.208
Time to achieve adequate sedation level	20.36±4.66	10.96±3.27 *	0.001
Time to achieve an Aldrete score of 10 (min)	40.53 ±6.51	37.60±6.42	0.084
Degree of patient’s satisfaction (a 7-point likert-like verbal rating scale)	6.46±0.62	5.56±1.04 *	0.023
Degree of surgeon’s satisfaction (a 7-point likert-like verbal rating scale)	5 76±0.97	5.35±1.33	0.081

Data are displayed as means±standard deviations.,

* Statistically significant compared to group D.

**Figure 4:**
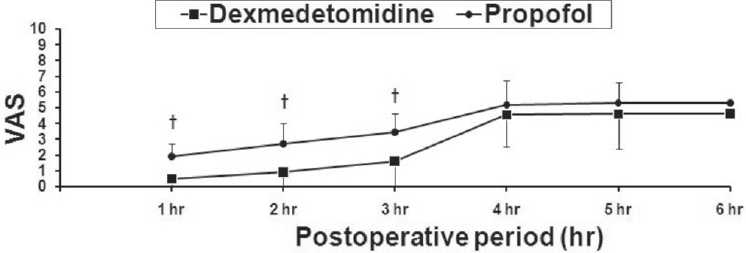
Visual analog scale for pain (VAS) during the postoperative period. Data are displayed as means±standard deviations. * Statistically significant compared to the baseline value. † Statistically significant compared to group D.

In the recovery room and postoperatively, we found that the time to achieve an Aldrete score of 10 were similar in both groups (*P*=0.084) [Table 2]. Dexmedetomidine has significantly lower VAS than propofol in the first3 h postoperatively [Figure 4]. The surgeon satisfaction with patients’ sedation was similar for both groups (*P*=0.081). While in the dexmedetomidine group, there was higher patients’ satisfaction compared with the propofol group (*P*=0.023) [Table 2].

## DISCUSSION

This study demonstrated that the use of dexmedetomidine at similar sedation levels with propofol during vitreoretinal surgery under local anesthesia was associated with equivalent hemodynamic effects, maintaining an adequate respiratory function, similar time of discharge from PACU, better analgesic properties, similar surgeon’s satisfaction with patient’s sedation and higher patient’s satisfaction.

The primary aim of this study was to compare the hemodynamic and respiratory effects in both groups. At similar sedative doses, dexmedetomidine and propofol resulted in a similar significant reduction in HR and MAP compared with baseline values. The same results were reported by Kaygusuz *et al*.[9] Previous studies had demonstrated a powerful inhibitory effect of propofol on sympathetic outflow.[10] Dexmedetomidine is also known to decrease sympathetic outflow and circulating catecholamine levels and would therefore be expected to cause decrease of MAP similar to those of propofol.[11] The decrease in the HR might be attributed to the sympatholytic effects and in part because of a vagal mimetic effect.[12]

Furthermore, the interesting finding in this study was that the dexmedetomidine sedation maintained an adequate respiratory function as compared with propofol sedation. The RR and SpaO_2_ values of the dexmedetomidine group were significantly higher than those in the propofol group during the sedation period. The expired CO_2_ was similar in both groups. Hsu *et al*.[13] reported similar effects on respiratory functions during dexmedetomidine sedation. They explained that by the increase in minute ventilation coincided with arousal phenomenon. Such arousal phenomenon, secondary to the hypercapnia stimulation, has been described during natural sleep. Dexmedetomidine converges on the natural sleep pathway to exert its sedative effects. Therefore, the similarity between the hypercapnic arousal phenomenon during dexmedetomidine infusions and natural sleep is not surprising. However, we reported that the expired CO_2_ was similar in both groups; the minute ventilation cannot be measured in the absence of endotracheal tube. Measurement of the expired CO_2_ through the nasal cannula is qualititative more than quantitative. In addition, De Sarro *et al*.[14] has reported that α-2 receptors are located at multiple places in the central nervous system. Hypercapnia activates the locus ceruleus, which is associated with increase apprehension and which leads to the stimulation of the respiratory centers. Ebert *et al*.,[10] also reported similar results with dexmedetomidine sedation.

On the other hand, Arain and Ebert[15] reported similar respiratory end points between dexmedetomidine and propofol groups while Kaygusuz *et al*.[9] reported that the RR values were significantly lower and the SpaO_2_ values were significantly higher in the dexmedetomidine group compared with the propofol group. This discrepancy in the results could be resulted from the difference in the regimen of drug infusion or the combination of narcotics. Sedative doses of propofol had been established to have minimal depressant effects on tidal volume and minute ventilation, with end-tidal CO_2_ tension and arterial blood gas values remaining unchanged.[16] In the recovery room and postoperatively, we found that the time to achieve an Aldrete score of 10 was similar in both groups and dexmedetomidine showed better analgesic properties than propofol (lower VAS) which was not relevant clinically as both groups had VAS scores<4. It is now well described that dexmedetomidine has analgesia sparing effects when used for sedation in the ICU.[17] The half-life of dexmedetomidine has been described as 2 h, would likely to explain why the analgesic sparing properties persisted postoperatively.[18] This interesting characteristic of dexmedetomidine (persistent analgesic effects with preserving the patient arousability) in the postoperative period resulted in significantly more analgesia with equivalent discharge times when compared with the short-acting propofol. Similar results were reported in a previous study by Arain and Ebert.[15]

Another finding in our study was that the surgeon’s satisfaction with patient’s sedation was similar for both groups. While, in the dexmedetomidine group, there was higher patient’s satisfaction compared with the propofol group which may be related to the natural sleep pathway of dexmedetomidine sedation. These results are similar to those reported by Arain and Ebert.[15]

In summary, monitored sedation for patients undergoing vitreoretinal surgery under sub-Tenon’s anesthesia is challenge for the anesthetist. Taking into account that benzodiazepines may result in respiratory depression and confusion, particularly, when administered to elderly patients,[3] and opioids are associated with increased risk of respiratory depression with episodes of apnea resulting in oxygen desaturation,[13] propofol is the widely used sedative hypnotic because of its minimal cardiorespiratoryeffects.[15,16] This study evaluated dexmedetomidine sedation, which is a highly selective alpha-2-adrenoreceptor agonist, compared with those of propofol sedation for this group of patients. We conclude that dexmedetomidine at a similar sedation levels with propofol was associated with equivalent hemodynamic effects, maintaining an adequate respiratory function, similar time of discharge from PACU, better analgesic properties, a similar surgeon’s satisfaction with patient’s sedation, and a higher patient’s satisfaction. Thus, dexmedetomidine may prove to be a valuable adjuvant for sedation in patients undergoing vitreoretinal surgery under sub-Tenon’s anesthesia.
